# Diagnosis of Delayed Cerebral Ischemia in Patients with Aneurysmal Subarachnoid Hemorrhage and Triggers for Intervention

**DOI:** 10.1007/s12028-023-01812-3

**Published:** 2023-08-03

**Authors:** Amr Abdulazim, Marina Heilig, Gabriel Rinkel, Nima Etminan

**Affiliations:** Department of Neurosurgery, Medical Faculty Mannheim, University Hospital Mannheim, University of Heidelberg, Theodor-Kutzer-Ufer 1-3, 68167 Mannheim, Germany

**Keywords:** Aneurysmal subarachnoid hemorrhage, Delayed cerebral ischemia, Multimodality monitoring, Triggers for intervention, Transcranial Doppler, Computed tomography perfusion, Continuous electroencephalography, Invasive monitoring

## Abstract

**Introduction:**

Delayed cerebral ischemia (DCI) is a major determinant for poor neurological outcome after aneurysmal subarachnoid hemorrhage (aSAH). Detection and treatment of DCI is a key component in the neurocritical care of patients with aSAH after initial aneurysm repair.

**Methods:**

Narrative review of the literature.

**Results:**

Over the past 2 decades, there has been a paradigm shift away from macrovascular (angiographic) vasospasm as a main diagnostic and therapeutic target. Instead, the pathophysiology of DCI is hypothesized to derive from several proischemic pathomechanisms. Clinical examination remains the most reliable means for monitoring and treatment of DCI, but its value is limited in comatose patients. In such patients, monitoring of DCI is usually based on numerous neurophysiological and/or radiological diagnostic modalities. Catheter angiography remains the gold standard for the detection of macrovascular spasm. Computed tomography (CT) angiography is increasingly used instead of catheter angiography because it is less invasive and may be combined with CT perfusion imaging. CT perfusion permits semiquantitative cerebral blood flow measurements, including the evaluation of the microcirculation. It may be used for prediction, early detection, and diagnosis of DCI, with yet-to-prove benefit on clinical outcome when used as a screening modality. Transcranial Doppler may be considered as an additional noninvasive screening tool for flow velocities in the middle cerebral artery, with limited accuracy in other cerebral arteries. Continuous electroencephalography enables detection of early signs of ischemia at a reversible stage prior to clinical manifestation. However, its widespread use is still limited because of the required infrastructure and expertise in data interpretation. Near-infrared spectroscopy, a noninvasive and continuous modality for evaluation of cerebral blood flow dynamics, has shown conflicting results and needs further validation. Monitoring techniques beyond neurological examinations may help in the detection of DCI, especially in comatose patients. However, these techniques are limited because of their invasive nature and/or restriction of measurements to focal brain areas.

**Conclusion:**

The current literature review underscores the need for incorporating existing modalities and developing new methods to evaluate brain perfusion, brain metabolism, and overall brain function more accurately and more globally.

## Introduction

Delayed cerebral ischemia (DCI) occurs in 30% of patients within the first 2 weeks after aneurysmal subarachnoid hemorrhage (aSAH). DCI is, apart from early brain injury, a major determinant for poor neurological outcome after aSAH [1, 2]. After initial aneurysm repair, the management of aSAH comprises prevention and detection of DCI as well as timely intervention to prevent cerebral infarction and thereby poor neurological outcome [3].

The following review will provide an overview on current modalities used for monitoring patients after aSAH, on the diagnosis of DCI, and on putative triggers for intervention.

## Definition of DCI

DCI is a clinical phenomenon, usually occurring 3–14 days after the primary hemorrhage, which may cause a delayed or secondary neurological deterioration (focal neurological deficit and/or decrease in level of consciousness). The diagnosis of neurological deterioration attributed to DCI is a diagnosis of exclusion, that is, other causes for neurological deterioration (such as infection, seizures, hydrocephalus, hypotension, metabolic disturbances, and/or postoperative), as well as postinterventional complications, should be ruled out [4, 5].

The nomenclature and definitions to describe the clinical phenomenon of DCI or radiological surrogates are often inconsistent and include terms such as delayed ischemic neurological deficit, delayed ischemic deficit, delayed neurologic deficit, secondary cerebral ischemia, vasospasm, clinical vasospasm, symptomatic vasospasm, symptomatic ischemia, and cerebral ischemia [4].

For consistency in reporting and comparability between clinical trials and observational studies, a multidisciplinary research group considered “delayed cerebral ischemia” to be the most appropriate term when reporting secondary neurological deterioration after aSAH that is not attributable to other causes and defined DCI as follows:

The occurrence of focal neurological impairment (such as hemiparesis, aphasia, apraxia, hemianopia, or neglect), or a decrease of at least 2 points on the Glasgow Coma Scale (either on the total score or on one of its individual components [eye, motor on either side, verbal]). This should last for at least 1 h, is not apparent immediately after aneurysm occlusion, and cannot be attributed to other causes by means of clinical assessment, CT or MRI scanning of the brain, and appropriate laboratory studies [4].

## Pathophysiology of DCI

Traditionally, it was assumed that DCI was predominantly caused by vasospasm visible on angiography. This concept, however, is challenged by the fact that only 30% of patients with aSAH develop clinical features of DCI, whereas 70% of them develop angiographic vasospasm [1, 6]. Further, up to 25% of delayed cerebral infarcts do not correlate with territories of arterial narrowing or may occur in patients without the evidence of vasospasm [7–9].

The Clazosentan to Overcome Neurological Ischemia and Infarction Occurring After Subarachnoid Hemorrhage trials demonstrated that clazosentan ameliorated vasospasm very effectively, but there was no beneficial effect on functional outcome [10–12]. In line with these findings, a meta-analysis of randomized controlled trials on the effect of pharmacological treatment on vasospasm, DCI, and clinical outcome underlined that angiographic vasospasm is not a major determinant nor a therapeutic target for functional outcome. Despite a reduction of vasospasm (risk reduction [RR] 0.80 [95% confidence interval (CI) 0.70–0.92]) in the pooled analysis of all experimental treatments, no statistically significant effect on poor outcome was observed (RR 0.93 [95% CI 0.85–1.03]) [13].

This understanding prompted a paradigm shift and led to further research into other possible pathophysiological causes. To date, the pathophysiology of DCI is still not fully understood and is hypothesized to be multifactorial, with an interaction of several proischemic pathomechanisms, such as angiographic vasospasm, microcirculatory dysfunction, microthromboembolism, cortical spreading ischemia, inflammation, and impaired cerebral autoregulation [14, 15]. This paradigm shift away from macrovascular (angiographically detectable) vasospasm as the main cause of cerebral ischemia and the main treatment target was additionally underlined by a meta-analysis of randomized controlled trials on medical prevention of DCI that had both cerebral infarction and functional outcome after subarachnoid hemorrhage (SAH) as outcome measurements. Pharmaceutical treatments that decreased the incidence of cerebral infarction (RR 0.83 [95% CI 0.74–0.93]) also reduced the risk of poor functional outcome (RR 0.92 [95% CI 0.86–0.98]) [3]. These data indicate that cerebral infarction is a more valid outcome measure than vasospasm in studies on pathomechanisms, prevention, and treatment of DCI.

## Risk Prediction of DCI

Although accurate prediction of DCI remains challenging, a sensitive tool for early detection of DCI would enable more efficient patient triage and risk-tailored monitoring strategies. Low-risk patients with SAH may then be monitored more liberally, while intensive monitoring and prophylactic measures, as well as invasive (rescue) treatment, may be focused on high-risk patients [16, 17]. Such a risk-stratified application of monitoring and treatment strategies may additionally avoid detrimental or adverse effects of invasive treatment, as well as improve cost-effectiveness, in the management of patients after SAH [14, 18].

The main risk factors for occurrence of DCI are the clinical condition on admission and the amount of SAH on the computed tomography (CT) scan [16, 17, 19, 20] (Tables 1 and 2). Other factors, such as smoking, diabetes, systemic inflammatory response syndrome, hyperglycemia, and hydrocephalus, have shown moderate to strong association with DCI [21].

**Table 1:** Modified Fisher Scale

Grade	Criteria	Risk for DCI
0	No SAH or IVH	
1	Focal or diffuse thin SAH, without IVH	24% (low)
2	Focal or diffuse thin SAH, with IVH	33% (intermediate)
3	Focal or diffuse thick SAH, without IVH	33% (intermediate)
4	Focal or diffuse thick SAH, with IVH	40% (high)

Adapted from Frontera et al. [19]

DCI, delayed cerebral ischemia, IVH, intraventricular hemorrhage, SAH, subarachnoid hemorrhage

**Table 2:** VASOGRADE

VASOGRADE	Criteria		LR +	LR −
	WFNS	mFS		
Green	1–2	1–2	1	–
Yellow	1–3	3–4	1.49	0.63
Red	4–5	1–5	1.94	0.68

Adapted from de Oliveira Manoel et al. [16]

DCI, delayed cerebral ischemia, LR, likelihood ratio, mFS, modified Fisher Scale, WFNS, World Federation of Neurological Surgeons

Research on cerebrospinal fluid molecules and serum biomarkers resulted in a number of markers associated with an increased risk for DCI and that might be useful in predicting DCI; however, none of these markers have been reproducibly validated for predicting the risk of DCI [22, 23].

Many studies have aimed to develop robust clinical and/or radiological grading scales to predict DCI or functional outcome and to potentially guide SAH treatment decisions [24]. However, most of these grading scales are not used in clinical practice because of their complexity and/or the required input data for risk prediction [25, 26]. More recently, the VASOGRADE, a simple three-category semiquantitative scale derived from pooled outcome data from previous SAH trials was developed as a practical grading system to predict the development of DCI [16] (Table 2).

## Diagnosis and Monitoring of Vasospasm and DCI: Available Evidence

Because of the importance of timely detection and the lack of sensitive biomarkers for DCI, there are numerous modalities and strategies for monitoring patients after aSAH to detect vasospasm (Table 3) and DCI (Table 4) and trigger intervention before the occurrence of cerebral infarction.

**Table 3:** Diagnostic modalities for vasospasm

Modality	Parameters of interest	Benefits	Limitations
TCD	Mild to moderate vasospasm: MFV 120–200 cm/s Lindegaard ratio 3–6 Severe vasospasm: MFV > 200 cm/s Lindegaard ratio > 6	Inexpensive, noninvasive, available at bedside, repeated measurements	Limited to detect large vessel vasospasm; poor interoperator reliability; application depends on available cranial bone window
DSA	Angiographic vasospasm: lumen narrowing > 70%	Gold standard	Invasive, limited availability
CTA	Angiographic vasospasm	Available, noninvasive	Less accurate for detecting small/medium vessel vasospasm; degree of angiographic vasospasm may be overestimated; artifacts from clips and coils

CTA, computed tomography angiography, DSA, digital subtraction angiography, MFV, mean flow velocities, TCD, transcranial Doppler ultrasonography

**Table 4:** Diagnostic modalities for DCI

Modality	Parameters of interest	Benefits	Limitations
Neurological examination	Alertness, speed of thinking Level of consciousness Pupillary size and reactiveness to light		Only possible in awake patients
CT perfusion imaging	MTT > 6.4 s or 1.5-fold prolongation compared to baseline indicative for DCI	Allows territorial evaluation of the cerebral microcirculation, detection of possibly salvageable brain tissue	Absolute cut off values are dependent on CT scanner and algorithm
EEG	ADR ↓ RAV ↓	Noninvasive, available, and continuous detection of ischemia at a possibly reversible state; exclusion of nonconvulsive status epilepticus	Requires software and experience
NIRS	rSO_2_	Noninvasive, continuous	Necessity of prospective validation
Invasive monitoring	CMD: LPR > 40, glucose < 0.7 mmol/l PtiO_2_ < 20 mm Hg Cerebral thermal diffusion flowmetry Cut off CBF: 15 ml/100 g/min	Measurement in the regional cerebral microcirculation at real time at bedside	Absolute cut off levels and implications for clinical treatment need to be further evaluated; only applicable in patients with poor-grade SAH; costs of equipment

ADR, alpha/delta ratio; CBF, cerebral blood flow; CMD, cerebral microdialysis; CT, computed tomography; DCI, delayed cerebral ischemia; EEG, electroencephalography; LPR, lactate/pyruvate ratio; MTT, mean transit time; NIRS, near-infrared spectroscopy; PtiO_2_, brain tissue oxygenation; RAV, relative alpha variability; rSO_2_, regional cerebral oxygen saturation; SAH, subarachnoid hemorrhage

## Vasospasm

### Concept of Vasospasm

Angiographic vasospasm is induced by a subarachnoid blood clot, and its severity is associated with the volume, density, location, and persistence of the blood clot encasing the subarachnoid arteries [27].

Angiographic vasospasm is mainly driven by hemoglobin and its hemolysis-mediated degradation products. This causes a multifactorial cascade of interacting pathophysiologic pathways (e.g., disrupted nitric oxide hemostasis, spreading depolarization, inflammation, and increased expression of endothelin-1), resulting in a reversal of the basal level of vasodilation toward vasospasm of both large and small arteries [14, 28].

However, it is strongly recommended to limit the use of terms such as “vasospasm” and/or “arterial narrowing” to the description of findings from radiological imaging [4].

### Transcranial Doppler

Transcranial Doppler ultrasonography (TCD) is an inexpensive and noninvasive modality with minimal risk used to measure blood flow velocities that reflect arterial diameters. TCD can reliably detect macrovascular spasm in the middle cerebral artery, but its accuracy for detecting macrovascular spasm in other cerebral arteries is rather poor [29]. Mean blood flow velocities of 120–200 cm/s are considered indicative of mild to moderate angiographic vasospasm, and mean blood flow velocities of > 200 cm/s are considered indicative of severe angiographic vasospasm [30, 31]. However, increased mean blood flow velocities may be the expression of physiologic or induced conditions, such as hyperemia and/or blood pressure augmentation [31]. The addition of the Lindegaard ratio (mean blood flow velocity of the middle cerebral artery divided by the mean blood flow velocity of the extracranial internal carotid artery) can improve the sensitivity to detect macrovascular spasm [32, 33]. The Lindegaard ratio indicates mild to moderate macrovascular spasm when the ratio is 3–6 and severe macrovascular spasm when the ratio is > 6 [31, 33].

Limitations of TCD are that it is an operator- and interpreter-dependent modality and that its application may be impeded by the available cranial bone window [14]. With respect to interpretation of TCD data, a drop from higher to lower velocities due to low blood flow may be misinterpreted as resolution of vasospasm [14].

In view of these limitations, we advise using TCD and interpreting TCD values in the context of clinical examination and additional monitoring modalities.

### Angiography

Catheter angiography remains the gold standard for detection of macrovascular spasm, but it is being increasingly replaced by CT angiography (which has an accuracy of up to 97.5% and negative predictive values of up to 99.5%) [34]. However, CT angiography is less accurate for assessment of medium and small vessels, and it may overestimate the degree of angiographic vasospasm [35]. Moreover, evaluation of macrovascular spasm using CT angiography may be limited because of beam-hardening artifacts from clips and coils [5, 14]. This limitation may at least be partly addressed by windowing and leveling of the CT scan, adjustment of the image reconstruction plane, and use of dual-energy CT scanners that enable more accurate visualization of the cerebral vasculature while reducing beam-hardening artifacts [36].

## DCI

### Clinical Examination

A thorough neurological examination constitutes the most reliable diagnostic means for monitoring and detecting DCI. Although DCI has been defined by clear clinical criteria, described above, the onset of DCI can be difficult to assess because DCI may start gradually or wax and wane with nonspecific signs, such as worsening headaches, confusion, or agitation. Apart from the insidious onset, two critical aspects further complicate the clinical detection of DCI. First, in comatose patients, neurological deterioration may not be reliably detected. Second, cerebral infarction may occur in patients with SAH in the absence of clinical symptoms for DCI. In fact, in studies in patients without clinical symptoms of DCI, CT and magnetic resonance imaging have revealed cerebral infarctions with similar patterns as DCI-related infarctions in 10–20% and 23% of patients, respectively [7, 37, 38].

Therefore, additional neurophysiological and/or radiological diagnostic tools are necessary to detect DCI and facilitate treatment, especially in patients who cannot be reliably neurologically assessed by serial neurological examinations [39, 40].

### CT Perfusion Imaging

Advanced imaging in the management of patients after aSAH includes CT perfusion, which is widely implemented in the monitoring and detection of DCI [41]. Through dynamic image acquisition following a contrast bolus, CT perfusion permits the territorial evaluation of the cerebral macrocirculation and microcirculation [42, 43]. Wintermark et al. [42] found that qualitative assessment of arterial narrowing on CT angiography in combination with a CT perfusion mean transit time (MTT) (the mean time it takes for blood to perfuse a region of tissue) of > 6.4 s had an accuracy of 93% for detecting DCI [42]. Ever since, CT perfusion has been widely implemented for prediction, early detection screening, and diagnosis of DCI [41, 44, 45]. Prolongation of the MTT has repeatedly been reported to be a sensitive parameter of compromised cerebral perfusion in the context of suspected DCI [43]. However, CT perfusion parameters and absolute cut off values are dependent on the CT scanner as well as on the algorithm used for measuring CT perfusion. Thus, absolute thresholds from published series cannot be always applied to other CT perfusion scanners or data sets [44]. Previous studies showed that a 1.5-fold prolongation of the MTT compared to baseline can be considered suggestive for DCI [46].

Compared to other modalities that may be considered in the assessment of cerebral perfusion, such as magnetic resonance perfusion, xenon-enhanced CT, and single-photon emission CT, CT perfusion is more readily available, pragmatic, and feasible, especially in critically ill patients [47].

Despite the disadvantages of radiation exposure and need for patient transportation, CT perfusion is estimated to have reasonable cost-effectiveness [48–50].

### Electroencephalography

Electroencephalography (EEG) constitutes a noninvasive, continuous, and real-time modality that enables monitoring and detection of seizure activity and/or ischemia, especially in patients with poor-grade aSAH, in whom neurological evaluation is of limited value [51]. Continuous EEG (cEEG) enables detection of early signs of ischemia at a reversible stage and prior to clinical correlates and/or findings in noncontinuous modalities (e.g., TCD and imaging modalities) [52]. Parameters such as a decreasing alpha/delta ratio, relative alpha variability, and total power derived from cEEG monitoring with quantitative EEG analysis have been shown to be indicative for DCI [53, 54]. When combined with TCD, the value of cEEG monitoring in the prediction of DCI increases [55]. Additionally, enhanced delta pattern, epileptiform activity, and nonconvulsive status epilepticus are associated with poor outcome and may facilitate prognostication by using cEEG monitoring [56, 57].

However, the costs for the required software and hardware, as well as the necessity for expertise to interpret the data, continue to limit the widespread use of cEEG monitoring in the management of patients after aSAH [35].

### NEAR-INFRARED Spectroscopy

Near-infrared spectroscopy (NIRS) is a continuous noninvasive modality that permits the evaluation of cerebral blood flow dynamics by estimating intracerebral oxygen saturation. NIRS has gained increasing interest in the monitoring of DCI [58]. A recent study in patients with high-grade SAH revealed a significant decrease in regional cerebral oxygen saturation in patients who developed DCI [58].

However, because there have been conflicting results on NIRS and DCI detection in the past, the value of NIRS should be prospectively validated in larger patient cohorts before this modality can be recommended for daily clinical practice [59].

### Invasive Monitoring

Invasive monitoring methods require the placement of a probe into a targeted brain region and allows for the focal measurement and evaluation of the brain microenvironment and metabolic state. Invasive monitoring includes microdialysis, brain tissue oxygenation, and thermal diffusion flowmetry.

Microdialysis probes may be used to detect changes in the concentrations of lactate, pyruvate, glucose, and glutamate. A lactate/pyruvate ratio of > 40 and brain glucose concentrations < 0.7 mmol/l are indicative of metabolic disturbance and a surrogate marker of ischemia [60]. In the context of multimodality monitoring after aSAH, microdialysis demonstrated metabolic distress prior to the manifestation of CT perfusion deficits and/or corresponding territory infarcts on CT [61, 62]. In a recent study, microdialysis allowed for an earlier detection of treatable DCI in patients with high-grade aSAH, and its use was associated with a reduction in overall DCI-related infarctions and unfavorable outcome [63].

Brain tissue oxygenation monitoring allows for continuous monitoring of brain tissue oxygen tension. The detection of lower cerebral tissue pH and higher partial pressure of carbon dioxide (pCO_2_) in patients with macrovascular spasms may serve as an indicator for the development of cerebral ischemia [64].

Cerebral thermal diffusion flowmetry provides continuous data on cerebral blood flow using an intraparenchymal probe [65]. A cut off value of 15 ml/100 g/min has been reported to have a sensitivity of 100% and a specificity of 75% for the detection of DCI [66].

Although all these techniques may provide additional information in the context of multimodality monitoring and may help in the early detection of DCI, major limitations include their invasive nature, cost, and the fact that monitoring is limited to the small region of the brain around the inserted probe [35].

## Triggers for Intervention

Although there has been effort in testing various clinical, neurophysiological, and radiological surrogates for subsequent development of DCI, there are no robust studies to test triggers for rescue therapy in such patients. This leads to significant practice variation between centers in the management of DCI. The recent Neurocritical Care Society guidelines for the neurocritical care management of aSAH concluded that there was insufficient evidence to provide a recommendation on the optimal trigger (change in neurological examination plus findings on advanced neuroimaging versus change in examination alone) for interventional procedures for the treatment of DCI [67].

## Practice Guidance

**Fig. 1:**
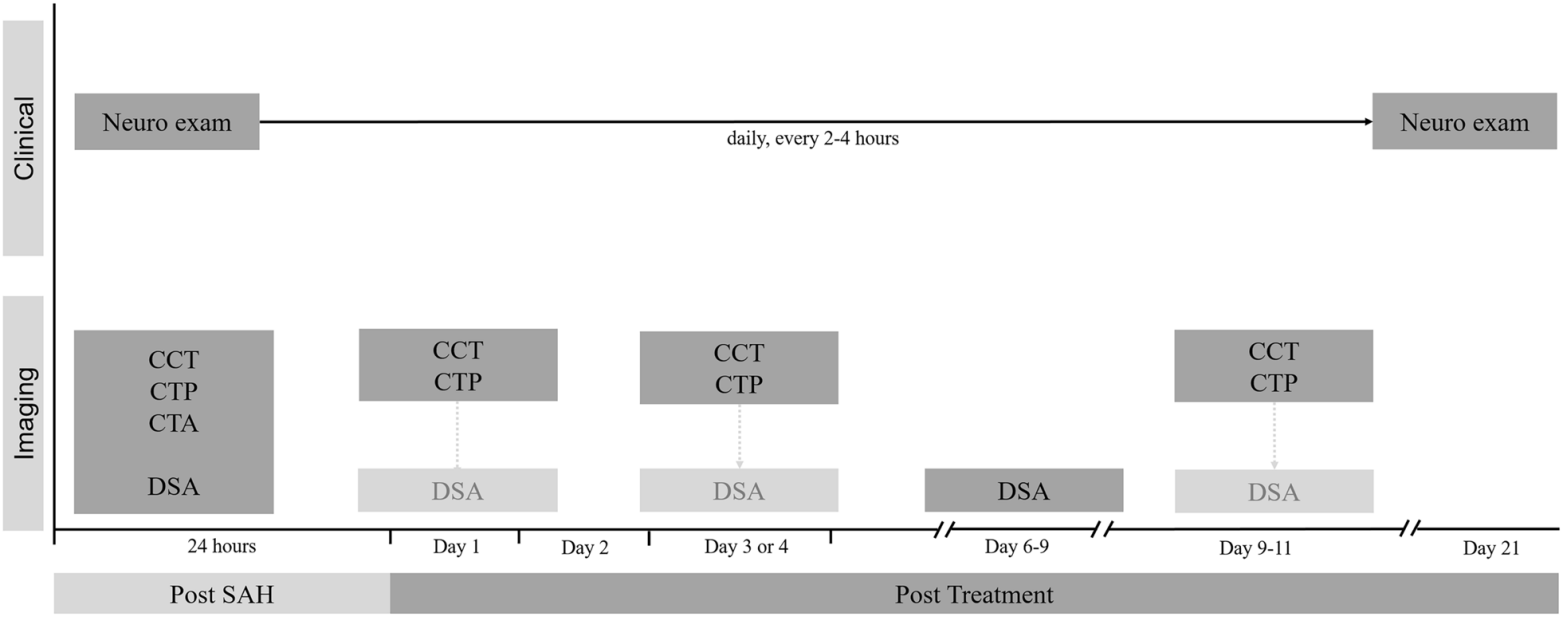
Standardized DCI monitoring protocol. The standardized monitoring protocol includes thorough neurological examinations every 2–4 h by dedicated neurocritical care staff in combination with a CT perfusion screening protocol. CT perfusion measurements are considered 6–12 h after aneurysm treatment, on day 3 or 4 as well as days 9–11 after aSAH, or in case of clinical features of DCI. DSAs are considered on admission and on days 6–9 after aSAH ictus and in case of clinical deterioration from DCI or CT perfusion deficits. CCT cranial computed tomography, CTA computed tomography angiography, CTP computed tomography perfusion, DSA digital subtraction angiography, Neuro exam neurological examination every 2 h, SAH subarachnoid hemorrhage. Adapted from Abdulazim et al. [50]

We recommend a rigorous and consistent approach to monitor patients with SAH that comprises standardized serial neurological examinations and imaging modalities to assess cerebrovascular perfusion or hemodynamics, especially in patients who cannot be reliably evaluated by neurological examination. In our practice, we perform CT perfusion imaging at predefined time points and when we have clinical suspicion of DCI but acknowledge that there is no good evidence proving that this strategy improves clinical outcome (Fig. 1). Additional means of monitoring (e.g., EEG monitoring, electrocorticography, NIRS, etc.) can be beneficial in comatose and/or high-risk patients, though they require infrastructure and experience in the interpretation of the information provided by these modalities. However, we want to underline that clinical monitoring for symptoms of DCI remains the strongest diagnostic method to detect DCI [68].

## Conclusions and Future Directions

The improving understanding on the pathogenesis of DCI has shifted the diagnostic and therapeutic target away from the macrovasculature toward the microvasculature and other mechanisms, including cortical spreading depolarization and inflammation. This underscores the need for incorporating existing modalities and developing new methods to evaluate brain perfusion, brain metabolism, and overall brain function more accurately and also more globally. Multimodality monitoring is advisable in comatose patients, but serial neurological examinations remain the mainstay of diagnosis of DCI in most patients with aSAH.

The lack of evidence for triggers of intervention and for interventions per se underlines the need for further studies addressing these pivotal topics. In this context and in view of the significant variability in practice, a cluster randomized clinical trial may provide more robust data.
